# Integrating Remote Sensing and Soil Features for Enhanced Machine Learning-Based Corn Yield Prediction in the Southern US

**DOI:** 10.3390/s25020543

**Published:** 2025-01-18

**Authors:** Sayantan Sarkar, Javier M. Osorio Leyton, Efrain Noa-Yarasca, Kabindra Adhikari, Chad B. Hajda, Douglas R. Smith

**Affiliations:** 1 Texas A&M AgriLife Blackland Research and Extension Center, Temple, TX 76502, USA; sayantan.sarkar@ag.tamu.edu (S.S.); efrain.noa-yarasca@ag.tamu.edu (E.N.-Y.); 2 United States Department of Agriculture–Agriculture Research Service, Grassland Soil and Water Research Laboratory, Temple, TX 76502, USA; kabindra.adhikari@usda.gov (K.A.); chad.hajda@usda.gov (C.B.H.); douglas.r.smith@usda.gov (D.R.S.)

**Keywords:** corn, maize, yield prediction, machine learning, vegetation indices, ensemble methods

## Abstract

Efficient and reliable corn (*Zea mays* L.) yield prediction is important for varietal selection by plant breeders and management decision-making by growers. Unlike prior studies that focus mainly on county-level or controlled laboratory-scale areas, this study targets a production-scale area, better representing real-world agricultural conditions and offering more practical relevance for farmers. Therefore, the objective of our study was to determine the best combination of vegetation indices and abiotic factors for predicting corn yield in a rain-fed, production-scale area, identify the most suitable corn growth stage for yield estimation using machine learning, and identify the most effective machine learning model for corn yield estimation. Our study used high-resolution (6 cm) aerial multispectral imagery. Sixty-two different predictors, including soil properties (sand, silt, and clay percentages), slope, spectral bands (red, green, blue, red-edge, NIR), vegetation indices (GNDRE, NDRE, TGI), color-space indices, and wavelengths were derived from the multispectral data collected at the seven (V4, V5, V6, V7, V9, V12, and V14/VT) growth stages of corn. Four regression and machine learning algorithms were evaluated for yield prediction: linear regression, random forest, extreme gradient boosting, and gradient boosting regressor. A total of 6865 yield values were used for model training and 1716 for validation. Results show that, using random forest method, the V14/VT stage had the best yield predictions (RMSE of 0.52 Mg/ha for a mean yield of 10.19 Mg/ha), and yield estimation at V6 stage was still feasible. We concluded that integrating abiotic factors, such as slope and soil properties, significantly improved model accuracy. Among vegetation indices, TGI, HUE, and GNDRE performed better. Results from this study can help farmers or crop consultants plan ahead for future logistics through enhanced early-season yield predictions and support farm profitability and sustainability.

## 1. Introduction

Accurate estimation of crop yields, particularly corn, is critical for efficient agricultural management, decision-making, resource allocation, crop insurance, and policy planning [1,2]. Corn (*Zea mays* L.) is an important crop, with a total production of 1.2 billion Mg over 197 million hectares globally. With a 30–33% share of global production, the USA is a top producer and exporter of corn [3,4]. Corn yield estimation plays a significant role in phenotyping for varietal selection, innovation of farm technologies, and influencing decisions on timing and logistics for harvesting, marketing, resource allocation, and risk management [5]. With the increasing variability in climate and the growing need for sustainable agricultural practices, the ability to predict yields accurately is more important than ever [6]. Accurate yield predictions help farmers and agricultural managers make informed decisions about crop management, irrigation scheduling, and the application of fertilizers and pesticides, leading to optimized use of resources, reduced costs, and improved crop yields [7]. Accurate yield estimation is crucial at different growth stages of corn, as each stage has distinct characteristics that affect yield outcomes. Corn growth stages include vegetative stages (emergence, leaf development, tasseling) and reproductive stages (silking, kernel development, maturity). The early- to mid-growth stages in the vegetative phase of plant development are critical for establishing the crop and provide the foundation for good yield [8]. Therefore, analyzing yield estimation accuracy at various early- to mid-growth stages can help identify the most suitable stage for making reliable yield predictions early in the season [9].

Traditional yield prediction methods, which include field sampling, manual counting, and visual inspection, largely depend on human observation and are subjective and spatially less accurate [9,10,11]. However, corn yield prediction is a complex challenge influenced by various factors, including weather, soil characteristics, and management practices. Research has demonstrated that integrating learning models with features derived from remote sensing can improve yield prediction [12,13]. Learning models are computational algorithms that recognize patterns in data to make predictions without being explicitly programmed. The application of learning models in agriculture has shown that using unoccupied aerial system (UAS)-based multispectral images and learning methods can effectively estimate physiological characteristics, such as chlorophyll content and leaf area index, leading to more accurate grain yield predictions [14,15]. Statistical methods provide powerful means for yield estimation as they can quantify complex relationships among high-dimensional input datasets that influence yield variations. Linear regression also known for its simplicity and ease of implementation, models the relationship between dependent and independent variables and has been widely used for yield estimation [16]. Nonetheless, its performance can be limited by its inability to capture non-linear relationships and interactions within the data. To address these limitations, machine learning and deep learning methods have emerged as effective alternatives. Machine learning methods, such as decision trees, work by recursively splitting the data into subsets based on the most important features, allowing them to model complex interactions between variables [17]. Deep learning methods employ artificial neural networks to automatically learn and extract features from large datasets, enabling advanced pattern recognition and decision-making. However, deep learning models typically require vast amounts of data—in the range of millions of data points—to perform effectively [17]. Therefore, decision trees are well-suited for agricultural problems because they handle mixed data types, interpret non-linear relationships, and are resilient to missing data, making them practical for analyzing diverse agricultural datasets.

Random forest (RF), a popular machine learning technique, constructs multiple decision trees during training and outputs the mode of the classes or the mean prediction of the individual trees [18]. This ensemble learning method is robust and effective in handling large datasets, making it suitable for yield estimation tasks [19]. EXtreme Gradient Boosting (XGBoost) is a decision tree ensemble regression algorithm that combines base functions with weights to enhance data fitting [20]. It demonstrates increased efficiency when dealing with large-scale datasets and complex models. The algorithm follows the gradient-boosting approach by iteratively training a series of weak learners (typically decision trees) to correct the residuals from the previous iteration. Gradient-boosting regressor (GBR) is an ensemble technique that, like RF, builds multiple decision trees to improve predictive accuracy [21,22]. GBR is particularly powerful in handling datasets with complex non-linear relationships but can be computationally demanding and sensitive to noise if not properly managed. However, parameter tuning of machine learning methods has often been rudimentary or unreported in recent studies, which can hamper the reproducibility of machine learning models [23,24,25].

Recent studies have demonstrated the potential of machine learning techniques and aerial imagery to estimate corn yields, achieving decent performance with error rates ranging from 5 to 8% using ensemble models [24,25,26,27]. Most of these studies were conducted under irrigated conditions and in cool climates, where planting is typically carried out around May. To our knowledge, there are no studies addressing corn yield estimation in the rain-fed conditions of the southern United States, where planting is carried out in February or March, and plants face heat and drought stress. A major issue with early planting is that corn does not accumulate enough Growing Degree Days (GDDs) during the early- and mid-season periods (125 and 480 GDDs instead of the recommended 520 and 1100 at V5 and V13, respectively) [28]. Additionally, previous studies have largely focused on describing the functionality of each machine learning model without explaining the performance of spectral properties and soil features or their correlation with corn growth and physiology. This gap in research has resulted in a lack of understanding as to why certain indices might be important for yield estimation. Moreover, only a few studies have focused on corn yield prediction in the early-growth stages, which is crucial for plant breeders in varietal selection and for growers in planning in-season nutrient applications, harvest, and post-harvest logistics [29,30]. Early developmental stages are particularly critical, as they can provide valuable indicators of a plant’s potential yield (in crops such as soybean, wheat, and sorghum) [31,32]. Furthermore, previous studies have only used spectral data or integration of multi-sensor indices and LiDAR [33]. Other factors, such as topography, elevation, and soil properties, also affect crop growth and development; therefore, the integration of spectral data could improve model accuracy [33,34]. Since plant growth and yield are determined by both genetic factors and environmental conditions, incorporating diverse data sources can help capture the complex interactions that affect plant performance [34,35].

As these technologies evolve, there is a growing consensus that such methods not only enhance prediction accuracy but also address the limitations of traditional methods [36]. One such method is use of vegetation indices (VIs) to quantify vegetation traits, including crop coefficient, leaf area index (LAI), chlorophyll content, fraction of absorbed photosynthetically active radiation, and biomass [14,15]. Multiple previous studies have adopted the normalized-difference vegetation index (NDVI) on a broad scale [37,38,39]. However, the past decade has seen the development of over 100 new spectral indices [40,41] due to the advent of affordable UAS 5-band multispectral sensors. For example, VIs such as the green normalized-difference vegetation index (GNDVI) [42], blue green pigment index (BGI) [43], modified chlorophyll absorption in reflectance index (MCARI) [44], structure-insensitive pigment index (SIPI) [45], and plant pigment ratio (PPR) [46] are sensitive to changes in leaf pigments (chlorophylls, xanthophyll, and carotenoids) and have shown significant potential in yield estimation and crop health assessment [47,48]. Sarkar et al. [40] and Balota et al. [49] have argued that VIs can be used as plant traits themselves, rather than just proxies for traits, given their heritability in plants. The authors, however, suggest that VIs are primarily influenced by leaf reflectance alone, a limitation that can be mitigated by complementing them with additional leaf spectral properties [50]. Apart from VIs, leaf color parameters, such as hue, saturation, and intensity, as well as inflection wavelengths, such as Red Edge Position (REP) and Red Edge Inflection Point (REIP), offer additional dimensions for assessing plant health [46,51,52]. Based on previous literature, there is a consensus that no single index is universally optimal, and integrating them with other remote sensing data improves accuracy in crop monitoring and yield predictions [36].

Along with spectral properties, other abiotic parameters, such as soil organic matter (OM), soil texture components, bulk density (BD), available water capacity (AWC), and topographical features like slope, have proven to be crucial predictors in crop yield forecasting [53,54,55]. Studies involving crops like soybean, wheat, and sorghum have demonstrated that combining these soil properties with VIs enhances crop yield predictions by capturing the complex interactions between crop physiology and environmental factors [53,54,55]. For instance, slope influences water drainage and erosion, directly affecting moisture availability and nutrient distribution across the field, which are critical for crop development [56,57]. OM, a key indicator of soil fertility, contributes to nutrient supply and water retention, which are essential for optimal plant growth and development [58,59]. Soil texture components—specifically, sand, silt, and clay—determine the soil’s ability to retain moisture and nutrients, impacting root development, and plant health. Sand improves aeration and drainage, while silt and clay enhance water and nutrient retention [60,61]. BD influences root penetration and water movement, while AWC indicates the soil’s ability to hold water that is accessible to plants, both of which are vital for maintaining plant health and yield during critical growth stages [62,63]. Recent studies have suggested that machine learning can help in selecting or combining indices in ways that traditional statistical methods might not, by considering multiple environmental and crop-specific factors simultaneously. They conclude that while NDVI remains popular, more complex indices (EVI, SAVI, etc.) or combinations can significantly improve monitoring capabilities, particularly when integrated with advanced analytical techniques [64,65]. However, research focused on estimating rain-fed corn yields using these models remains limited. Additionally, most existing studies have concentrated on county-level or experiment-scale areas. In contrast, this study focuses on a production-scale area (field scale), which more accurately represents real-world agricultural conditions and provides greater relevance for farmers. Furthermore, the integration of physical terrain characteristics and imagery data with machine learning models in rain-fed, production-scale corn fields is still an underexplored area of research.

In this regard, after an extensive literature review, it became clear that studies integrating soil features with spectral and topographic data for corn yield prediction are limited, despite the crucial role of soil variables in agricultural productivity. Recent works have focused on yield prediction at the pixel level within field-scale using satellite imagery, occasionally incorporating topographic and genotypic data [60,66,67,68,69,70,71]. However, these studies overlook the critical role of soil [72,73]. Studies including soil properties show potential but also leave gaps. For example, Khanal et al. [74] identified soil class as a key predictor, using variables like organic matter, cation exchange capacity, and pH, but lacked specificity in linking these to the corn yield. Xu et al. [75] used two soil attributes (available water capacity and organic matter) from the SSURGO database, while broader studies by Dhaliwal and Williams [73] and Shahhosseini et al. [76] incorporated soil texture, bulk density, and water retention capacity but excluded spectral data. These gaps highlight the need for more comprehensive models integrating soil, spectral, and topographic data to improve yield prediction accuracy.

In this context, this study presents an innovative approach that integrates spectral data from remote sensors, terrain information from digital elevation models (DEMs), and soil properties from the POLARIS database. Compared to SSURGO, POLARIS offers more uniform data and critical soil variables, even in areas lacking SSURGO data [77,78]. By combining these datasets, the approach addresses key limitations in yield prediction, particularly at fine spatial scales, such as the farm level. This study demonstrates how integrating spectral data from remote sensing, topographic data, and soil properties with machine learning models can enhance corn yield prediction. This approach offers a more reliable framework for agricultural decision-making and supports sustainable resource management. Our objectives are: (i) to identify the optimal combination of vegetation indices (VIs), soil properties, and topographic features for predicting corn yield in a rain-fed, production-scale area; (ii) to determine the most suitable corn growth stage for yield estimation using machine learning; and (iii) to evaluate the performance of various machine learning models for corn yield prediction.

## 2. Materials and Methods

This Section outlines the methods and materials used to assess corn yield in a real-world agricultural setting. The study site is described in terms of location, soil characteristics, and management practices. Key data collection procedures include drone-based multispectral image acquisition, radiometric calibration, and yield monitoring with GPS integration. Soil, topographic, and spectral data were processed to extract relevant predictors for statistical and machine-learning-based yield modeling. Four regression models—multiple linear regression (MLR), random forest (RF), gradient-boosting regressor (GBR), and XGBoost—were employed to predict yield, with a focus on evaluating the accuracy and applicability of these methods.

### 2.1. Location

**Figure 1:**
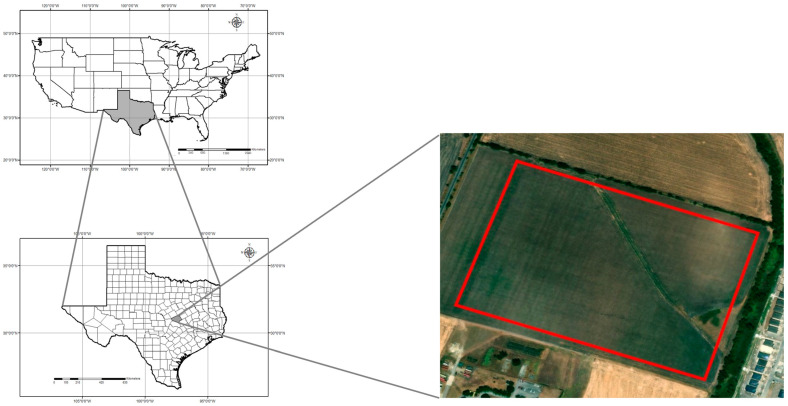
Map of the study area and location of the experimental site. The two maps on the left indicate the U.S.A. and the state of Texas; a grayed area indicates Bell County. The map on the right indicates the field study site at the AgriLife-Blackland Research and Extension center in Temple, Texas.

**Figure 2:**
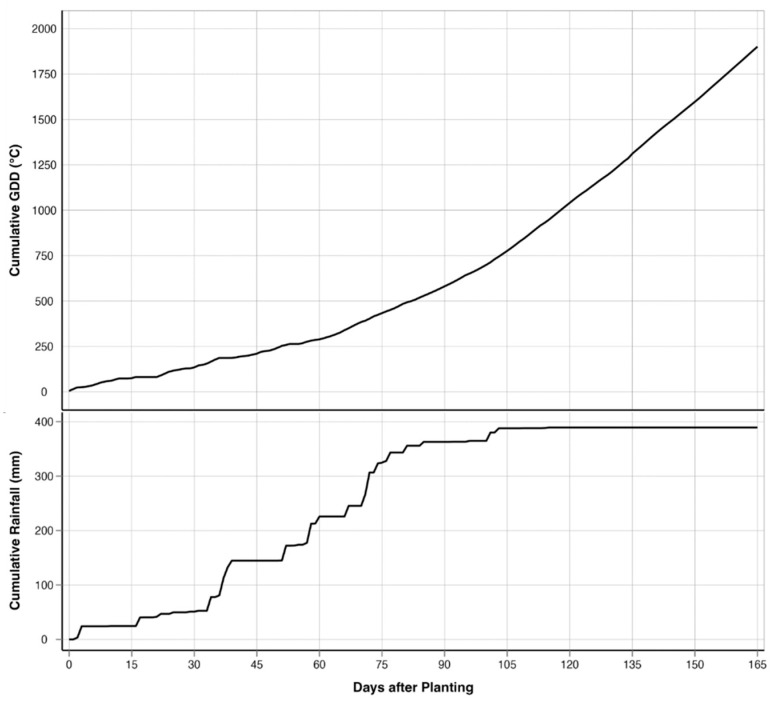
Progression of cumulative precipitation (PRCP) and cumulative growing degree days (GDDs) over the growing period.

This study was conducted in a 19.03-ha rain-fed field cooperatively managed by Texas A&M AgriLife’s Blackland Research and Extension Center and the USDA-ARS’s Grassland, Soil and Water Research Laboratory in Temple, Texas (31.059444° N; 97.345833° W; 192 m elevation) throughout the spring and summer of 2023 (Figure 1). The field was planted with corn on 28 February 2023 and was fertilized with 448 kg/ha of 32.5N - 16.2P - 0K - 1.2S - 1.5Zn broadcasted before planting. The corn was seeded at 4.8 plants/m^2^ using no-till practices, resulting in a planting density of 68,918 seeds/ha. The corn was harvested on 14 August 2023. The study area is in the Blackland Prairies ecoregion, and the soils are a mix of Houston Black clay (fine, smectitic, thermic, Udic Haplusterts) and Austin clay (fine-silty, carbonatic, thermic Udorthentic Haplustolls) [79]. These heavy clay soils are rich in nutrients such as calcium and magnesium but tend to compact easily, making root penetration and water infiltration challenging. The cumulative rainfall during the growing season was 389.4 mm, and the cumulative GDD was 1929 °C for over 167 days from planting to harvest (Figure 2). The field has a relatively flat topography, with a gradual elevation change of 8 m between the highest and lowest points. The highest point is located diagonally in the center of the field, forming a slight ridge. From this point, the terrain gently slopes downward toward the northeast and southwest corners, creating a subtle but noticeable elevation gradient. Despite the overall slope, the surface still allows for slight variations in drainage across the field.

The corn was harvested using a John Deere 9510 combine (Deere and Company, Moline, IL, USA), and yield was recorded in real time by the Ag Leader corn yield monitor (Ag Leader Technology Inc., Ames, IA, USA). The yield monitor recorded the yield of six rows (covering an area of 16.9 m^2^) of corn along with the coordinates of the centroid of the harvested area using an in-built GPS recorder. Pre-harvest and in-field calibration of the yield monitor was conducted for load size, weighing, and GPS accuracy. Raw yield data were pre-processed using Yield Editor software 2.7.0 (USDA-ARS, Columbia, MO, USA) [80] for speed and pass delays, overlap and moisture corrections, and potential outlier detection and removal [81]. The cleaned yield values were used as ground truth data for our model training and testing, and the corresponding GPS locations were used to create polygon shapefiles for feature extraction.

Our study was conducted in a 20 ha rain-fed field with a history of no-till management and minimal disturbances, as opposed to controlled experimental plots, and without the structure of a formal experimental design. This approach allowed us to observe real-world agricultural conditions, providing results more directly applicable to everyday farming scenarios. By conducting the research in a typical farming environment, we aimed to capture variability and challenges that may not be present in controlled experiments, making our findings more relevant to actual farming practices. However, the lack of a formal experimental design means that our results may be influenced by uncontrolled variables inherent to the field setting.

### 2.2. Multispectral Image Acquisition

We flew a WingtraOne GenII fixed-wing drone (Wingtra, Zurich, Switzerland) equipped with a MicaSense RedEdge-P multispectral sensor (AgEagle Aerial Systems, Seattle, WA, USA). The sensor has five multispectral bands: blue (459–491 nm), green (546.5–573.5 nm), red (661–675 nm), red-edge (711–723 nm), and near-infrared (813.5–870.5 nm), each with a resolution of 1456 × 1088 (1.6 MP). The mission-planning process was performed using the WingtraHub 1.0 software (Wingtra, Zurich, Switzerland) in the UAS controller. Flight operations were conducted autonomously using pre-defined routes seven times corresponding to various vegetative growth stages (Table 1). The individual images were collected at an altitude of 60 m above the ground level (AGL) with forward and sideways overlap of 75%, resulting in a ground sampling distance (GSD) of 6 cm. All flights were conducted between 11:30 AM and 12:30 PM to ensure uniformity in data collection, minimize shadows, and provide consistent illumination. The built-in GPS of the UAS was used for flight navigation, nadir image acquisition, and recording coordinates of individual images. For each flight, we maintained the same camera settings and employed the same mission planning technique to guarantee our model’s comparability, accuracy, and consistency. Geospatial corrections for images were also conducted in WingtraHub using post-processing kinematics (PPK). Rinex files for PPK were generated using Reach RS2+ Multi-Band RTK GNSS Receiver (Emlid Tech Kft., Esztergomi, Budapest). The collected images were then orthomosaiced in Pix4Dmapper Version 4.8.4 software (Prilly, Switzerland) to create multispectral rasters in all five bands.

### 2.3. Radiometric Calibration

The MicaSense RedEdge-P multispectral camera incorporates an advanced radiometric calibration workflow to ensure accurate spectral measurements. At the beginning and at the end of the image acquisition, we used a calibrated reflectance panel (CRP) to capture reference images under the same lighting conditions as the target scene. Additionally, the camera’s internal calibration system compensates for factors, such as lens transmittance, optical vignetting, and sensor-specific characteristics like quantum efficiency and non-linear response. The acquired images are then processed using Pix4D Mapper 4.8.3 software (Pix4D, Prilly, Switzerland), which utilizes CRP data to apply radiometric corrections for variations in illumination and atmospheric conditions, producing reflectance-calibrated multispectral outputs.

### 2.4. Soils and Topographic Data

Soil property raster files were obtained from the POLARIS database, maintained by Duke University (http://hydrology.cee.duke.edu/POLARIS/, accessed on 21 August 2024) [69,70]. The downloaded rasters included data on sand percentage, silt percentage, clay percentage, OM, BD, and AWC. These soil raster layers, originally at a 30-m resolution, were resampled to a 1-meter pixel size to ensure compatibility with the spatial resolution of the images collected with the drone. The study area exhibits a distinct separation between Houston Black and Austin soils, which are clearly identifiable. We verified that the original resolution of the soil map adequately captures this variation. We used the nearest-neighbor method to preserve the original data. This method decreases spatial resolution without modifying the pixel values, but it does not introduce any new information. The Digital Elevation Model (DEM; 1m) raster was downloaded from publicly available LiDAR data provided by United States Department of Agriculture Natural Resource Conservation Service. Because the slope variability across the area was relatively gradual and did not display any sudden or significant changes in elevation, we decided to use a 1-meter digital elevation model which adequately captures the variation in slope. The DEM’s resolution provided reliable terrain representation without sacrificing accuracy, as the terrain did not contain sharp inclines or declines that would require higher precision data. A raster for the slope of the study area was derived from the DEM raster using the ‘Slope’ tool of ArcGIS Pro (version 3.3.0) (ESRI, Redlands, CA, USA).

### 2.5. Feature Extraction

For feature extraction, the red, green, blue, red-edge, and NIR reflectance rasters, along with slope and soil property (sand, silt, clay, OM, BD, and AWC) rasters, were exported to ArcGIS Pro. To extract spatial information on vegetation from reflectance rasters, bare ground masking was carried out to remove soil pixels. The masking was performed using the normalized difference vegetation index red edge (NDVIre) raster. The threshold value for masking was determined manually for each flight. Pixels with NDVIre values less than the threshold value were soil, and the remaining were vegetation. A binary raster (1 for vegetation and 0 for soil) was created, and all five orthomosaiced rasters were multiplied by the binary raster to remove soil pixels. This process was repeated for each flight date. Zonal statistics of each of the rasters were extracted using the polygon shapefile to obtain an average value of raster pixels within each polygon. Since the pixel values within each polygon were averaged, the number of data points were similar for 6 cm resolution spectral bands and 1 m resolution soil and slope raster. The reflectance values from extracted statistics were used to calculate vegetation indices (VIs), color space indices (CSIs), and wavelength values (WVs) (Table 2). Hue, saturation, and intensity are CSIs belonging to hue, saturation, and intensity (HIS) color space; REP and REIP are wavelength values; and rest of the derivations are VIs. All the spectral data including individual bands and soil, and topographical data were used as predictors for initial model training. To extract spatial information from all rasters corresponding to ground truth data, polygon shapefiles were created from GPS points received from the harvester corresponding to corn yields. The GPS points were imported as shapefile over ArcGIS Pro and Thiessen polygons were created. Irregular polygons were weeded out by applying thresholds based on area and circularity index. Polygons on the edges were removed to minimize the bordering effect. A total of 8581 polygons were finally present in the shapefile. The average roundness index of the polygons was 2.44 ± 0.08, which shows that they were very uniform in shape. The average area of the polygons was 16.89 ± 1.64 m^2^, and the perimeter was 16.04 ± 0.71 m. The polygons were uniformly distributed across our field of study. It was made sure that the polygons comprehensively covered the reflectance, soil, and slope rasters.

### 2.6. Statistical and Machine Learning Models

Four regression models, multiple linear regression (MLR), RF, XGBoost, and GBR, were used. The regression analysis was performed using Python 3.12.

MLR: The MLR model was used as a baseline in this study. It models the relationship between a dependent variable Y and multiple independent variables $X_{1},\;X_{2},\;\ldots ,\;X_{n}$ as follows:

$$Y=\beta_{0}+\sum_{i=1}^{n}\beta_{i}\;X_{i}+\epsilon$$

where $\beta_{0}$ is the intercept, $\beta_{i}$ represents the coefficients, and *ϵ* is the error term, capturing the variation in Y that is not explained by the independent variables. The coefficients are estimated to minimize the difference between observed and predicted values.

RF: RF is an ensemble learning method used for classification and regression tasks, which operates by constructing a multitude of decision trees during training. “Random” refers to the process of growing the decision trees, with each tree in the RF being distinct from the others. In RF, each decision tree is trained independently on randomly selected subsets, thus reducing the risk of overfitting. The method aggregates the predictions of individual trees to improve overall accuracy and reduce overfitting [82]. In the context of regression, the prediction is computed as the average of the predictions from all individual trees:

$$\hat{Y}=\frac{1}{T}\sum_{t=1}^{T}{\hat{Y}}_{t}$$

where ${\hat{Y}}_{t}$ is the prediction from the *t*-th decision tree, and T is the total number of trees. The RF method is particularly robust because it introduces randomness by selecting a random subset of features for each split in the trees, enhancing model generalization and performance. Moreover, the construction of each decision tree is guided by minimizing the mean squared error (MSE) at each node, which helps in identifying the most informative features while reducing bias. The random selection of both data samples and features not only mitigates the risk of overfitting but also improves the model’s ability to handle complex datasets with high dimensionality [83,84].

GBR: GBR is an ensemble learning method used for regression tasks that builds a series of weak predictive models, typically decision trees, in a sequential manner. Each new tree is trained to correct the errors made by the previous ones, with the goal of minimizing the overall prediction error [85]. The method optimizes a loss function by iteratively adding models to the ensemble:

$$\hat{Y}=\sum_{m=1}^{M}v\;h_{m}(X)$$

where $\hat{Y}$ is the predicted value, M is the total number of trees, $h_{m}(X)$ is the prediction from the *m*-th tree, and v (the learning rate) controls the contribution of each tree. GBR is highly effective for capturing complex patterns in the data, making it a powerful tool for predictive modeling. It is particularly powerful in handling datasets with complex non-linear relationships but can be computationally demanding and sensitive to noise if not properly managed. GBR employs a technique called gradient descent to minimize the loss function, allowing for continuous improvement of model accuracy. This method not only enhances predictive performance but also enables the identification of key predictors through its ability to manage interactions and non-linear relationships among variables [85].

XGBoost: XGBoost is an advanced implementation of the gradient-boosting technique. It builds an ensemble of decision trees in a sequential manner, where each tree attempts to correct the errors made by the previous ones. The algorithm follows the gradient-boosting approach by iteratively training a series of weak learners (typically decision trees) to correct the residuals from the previous iteration. Iterations continually enhance the overall performance of the model, ultimately combining these weak learners into strong learners. XGBoost is known for its high accuracy and robustness against overfitting, but it requires careful tuning of hyperparameters to achieve optimal performance. XGBoost incorporates several optimizations, such as regularization, parallel processing, and handling missing data, which enhance its performance and scalability [51,86,87]. The model prediction is given by the following Equation:

$$\hat{Y}=\sum_{m=1}^{M}h_{m}(X)$$

where $h_{m}(X)$ represents the prediction from the *m*-th tree, and M is the total number of trees. The objective function that XGBoost minimizes consists of both the loss function L and a regularization term Ω to control model complexity:

$$Obj=\sum_{i=1}^{n}L(y_{i},{\hat{y}}_{i})+\sum_{m=1}^{M}\left(h_{m}\right)$$

where $L(y_{i},{\hat{y}}_{i})$ is the loss function measuring the difference between the true and predicted values, and $\Omega (h_{m})$ is the regularization term that penalizes overly complex models. XGBoost is known for its efficiency, speed, and accuracy, making it a popular choice in machine learning competitions and practical applications [86]. In addition to its efficient handling of large datasets, XGBoost employs a unique tree-pruning mechanism that optimizes the tree structure, thereby improving generalization. Its built-in cross-validation feature facilitates hyperparameter tuning, ensuring robust model performance across diverse applications, particularly in remote sensing [51,86,87,88].

### 2.7. Hyperparameters Tuning and Data Training

To achieve accurate predictions, hyperparameters for each model were carefully optimized using grid search within practical limits. This well-established technique, implemented in Python with libraries such as NumPy [89], Pandas [90], and Scikit-learn [91], involves systematically exploring a range of parameter values, training the model across all possible combinations, and selecting the best-performing settings.

- RF: ‘max_depth’: 21, ‘max_features’: 11, ‘n_estimators’: 500
- XGBoost: ‘colsample_bytree’: 0.9, ‘gamma’: 0.30, ‘learning_rate’: 0.05, ‘max_depth’: 6, ‘n_estimators’: 200, ‘n_jobs’: 8, ‘subsample’: 0.7, ‘verbosity’: 1
- GBR: ‘learning_rate’: 0.1, ‘max_depth’: 5, ‘n_estimators’: 200, ‘subsample’: 0.7

**Figure 3:**
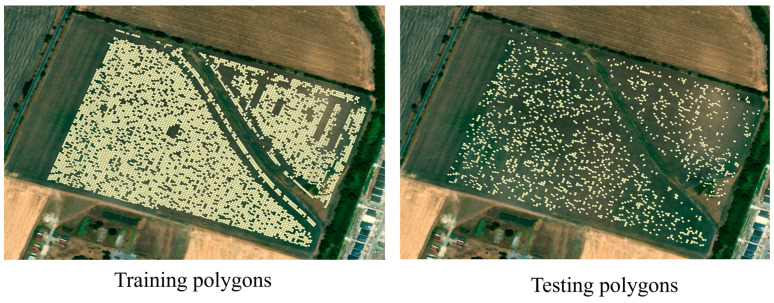
Spatial distribution of training and testing samples.

The dataset was randomly divided into training and testing subsets using an 80:20 ratio, yielding 6865 samples for training and 1716 for testing (Figure 3). The following optimal hyperparameters were identified for each model:

### 2.8. Model Performance Evaluation

The model accuracy was evaluated through the computation of the coefficient of determination (R^2^) and root mean square error (RMSE) values. The regression analysis calculated the R^2^ values for the VIs as independent or predictor variables, and yield data as the dependent or predicted variables. The increased predictability of the predicted variables from the predictor variable can be determined by higher R^2^ and lower RMSE values. Consequently, R^2^ and RMSE were determined using Equations (1) and (2), respectively.

(1) $$R^{2}=1-\frac{\sum_{i=1}^{n}{\left(y_{i}-\hat{y_{i}}\right)}^{2}}{\sum_{i=1}^{n}{\left(y_{i}-\bar{y}\right)}^{2}}$$

where $y_{i}\;$ represents the observed values; $\hat{y_{i}}$ represents the predicted values; $\bar{y}$ is the mean of the observed values; and n is the number of observations.

(2) $$\text{RMSE}=\sqrt{\frac{1}{n}\sum_{i=1}^{n}{\left(y_{i}-\hat{y_{i}}\right)}^{2}}$$

where $y_{i}\;$ represents the observed values; $\hat{y_{i}}$ represents the predicted values; and n is the number of observations.

The predictors were further tested for multicollinearity, and their feature importance was determined. Predictors with lower feature importance and those that were collinear were removed, and the models were tested again. This process was repeated until an optimal number of predictors for each growth stage was obtained. Models with the fewest predictors and lowest RMSEs for the testing dataset were finalized. Additionally, analysis of variation (ANOVA) and mean separation of RMSEs across regression methods were conducted using Fisher’s least significant difference (LSD) to compare the accuracies. Further, the soil and elevation data were removed from the final set of predictors and the ensemble methods (RF, XGBoost, and GBR) were tested again. The RMSE of these models were compared with those of full models using a one-tailed *t*-test.

## 3. Results and Discussion

### 3.1. Measured Yield Variable

The yield measurements recorded with the built-in monitor (n = 8551) provided the following statistics. The minimum and maximum yield values for our study are 1.69 Mg/ha and 15.86 Mg/ha, respectively. The first quartile (Q1) has a value of 9.46 Mg/ha, while the third quartile (Q3) has a value of 10.95 Mg/ha, with an interquartile range (Q3–Q1) of 1.49 Mg/ha. The average yield was 10.19 Mg/ha, higher than the average yield in Texas for the 2022/2023 harvest season, which was 6.62 Mg/ha [4]. The coefficient of variation (CV) was 12.11%, which implies that yields were consistent across the study area. The interquartile range indicates that most of the yield data are clustered closely around the median, indicating moderate variability and allowing for reliable estimation.

### 3.2. Prediction Methods

**Figure 4:**
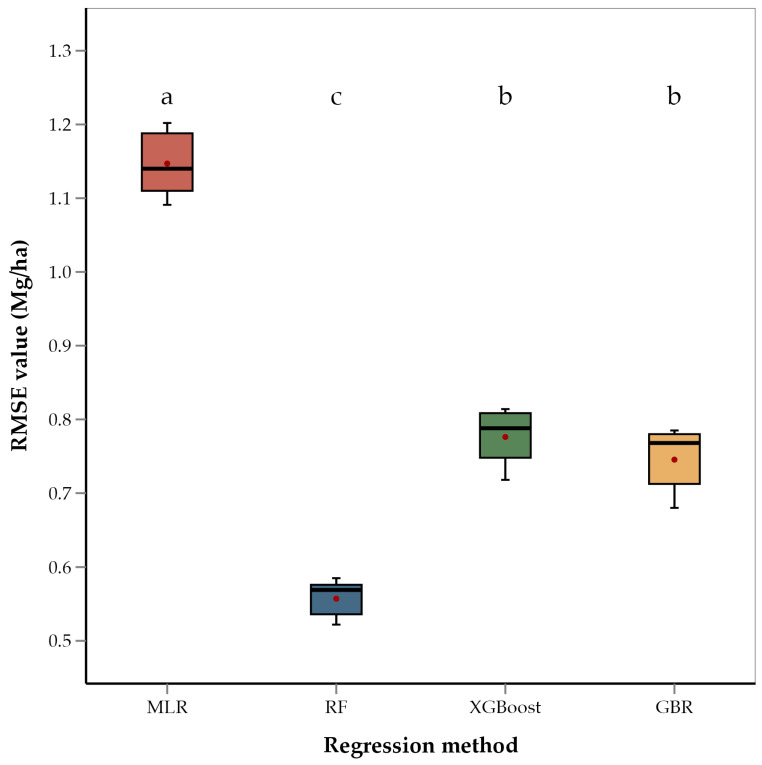
Box and whisker plot of average RMSE values of regression methods used. Box plots with different letters are significantly different (‘a’ being the highest and ‘c’ the lowest) using Fisher’s protected LSD at α = 0.05.

ANOVA of RMSEs across the four regression methods showed that the methods differed significantly (*p* < 0.001). Mean separation using Fishers’ LSD showed that RF had the lowest average RMSE value (0.56 Mg/ha) of the four methods (Figure 4). This is consistent with several recent studies that have used RF for yield estimation of crops such as rice and cotton and have found RF to be better performing than GBR and XGBoost [92,93,94,95]. These studies suggest that the superior performance of RF is due to its robust ability to handle large datasets and effectively model complex, non-linear interactions among predictor variables. RF constructs multiple decision trees using random subsets of data and features, which not only reduces the risk of overfitting but also improves the generalization of the model to new data [87,93]. Unlike other models that may focus heavily on specific features or data subsets, RF’s use of bootstrapping and the aggregation of predictions from multiple decision trees provides a more balanced approach to yield prediction, capturing diverse aspects of the data and thereby enhancing accuracy and robustness [96]. This robustness is particularly advantageous in agricultural settings where data can be noisy and variable. Recent literature supports the use of tree-based ensemble methods like RF, XGBoost, and GBR for yield prediction in place of linear models [97,98]. These studies have shown that tree-based models outperform linear models in predicting crop yields. Ensemble models penalize the incorrect output while training the model and try to minimize the loss, highlighting their capacity to model non-linear relationships and interactions among environmental variables. Therefore, the relatively poorer performance of the MLR model in our study is consistent with the model’s limitations in handling non-linear relationships and interactions between features. The performance of XGBoost, while better than MLR, was not as strong as that of GBR and RF. This fact could be attributed to the sensitivity of XGBoost to hyperparameter settings and overfitting in the case of noisy or heterogenous data [99]. XGBoost is known for its efficiency and high performance, particularly in structured data, but it may require extensive tuning to achieve optimal results.

### 3.3. Optimum Growth Stage

**Figure 5:**
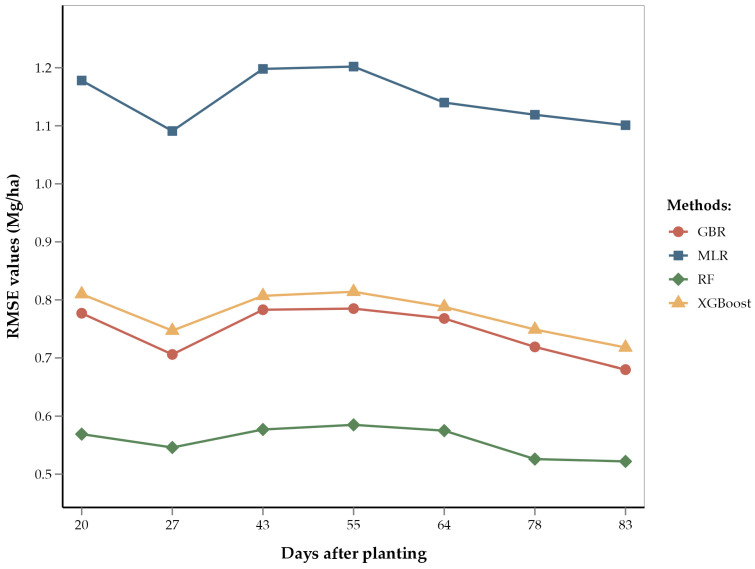
Line diagram showing the RMSE of different regression methods by days after planting.

According to our results, the V12 (12-leaf) through V14/VT (14-leaf/tasseling) stages have the lowest RMSE values (0.52 Mg/ha) for the RF model as compared to other growth stages (Figure 5). This shows that yield predictions are most accurate during late vegetative stages. These findings are consistent with previous studies, which concluded that remote sensing data collected during the tasseling stage provide reliable yield predictions [9,23]. The improved accuracy of yield estimation during late vegetative stages can be attributed to the corn growth physiology during these stages. The 12- to 14-leaf stages correspond to peak vegetative growth and consequently involve significant physiological activities, such as photosynthate and starch accumulation for future corn kernels [100]. The tasseling stage marks the transition from vegetative growth to the reproductive stage. During this stage, the corn plant is focused on pollen formation and pollination [101]. Therefore, a healthy VT stage and higher pollination rate leads to better seed formation. These physiological processes during the end of the vegetative phase are crucial for cob growth and seed yield.

Our results also show that early-season corn yield estimates at the V5 (5-leaf) stage were almost as accurate (RMSE = 0.54 Mg/ha) as estimates at the V12 through V14/VT stages. This can be attributed to the early corn growth physiology, which significantly influences vegetative phase development [101]. During the 5-leaf stage, healthy corn plants engage in robust photosynthesis, leading to increased leaf formation. This stage is foundational for the plant’s overall growth, determining the plant’s potential to reach the 14-leaf stage (optimum for our study) [28]. At these early stages, plants are highly responsive to growth inputs such as water and nutrients. Adequate provision of these inputs ensures vigorous growth and optimal leaf area development, which is essential for maximum light interception and photosynthate production. Conversely, stress or deficiency during this stage results in stunted growth, reduced leaf numbers, and lower photosynthate allocation to the sink (developing corn cob in this case) [102].

Although yield estimates at the V5 and V12 through V14/VT stages were most accurate, estimates at other vegetative stages demonstrated comparable accuracy. The RMSEs of the RF model ranged from 0.52 to 0.58 Mg/ha (i.e., errors ranging from 5.1% to 5.7%). This relatively narrow range of errors can be attributed to the large number of data points (6865 training points) used in the model; these numerous data points capture variation and heterogeneity, thereby improving model accuracy [102,103]. Given the accuracy and reliability of yield estimations during both the early and late vegetative stages, we recommend optimizing future remote sensing flights to focus on the V5 and V14/VT stages. This targeted approach reduces operational costs and improves data accuracy by focusing on critical growth periods. Accurate data collected at these stages can help in early-season monitoring and management decisions, ensuring that plants are on track for optimal growth.

### 3.4. Soil Properties and Slope

**Figure 6:**
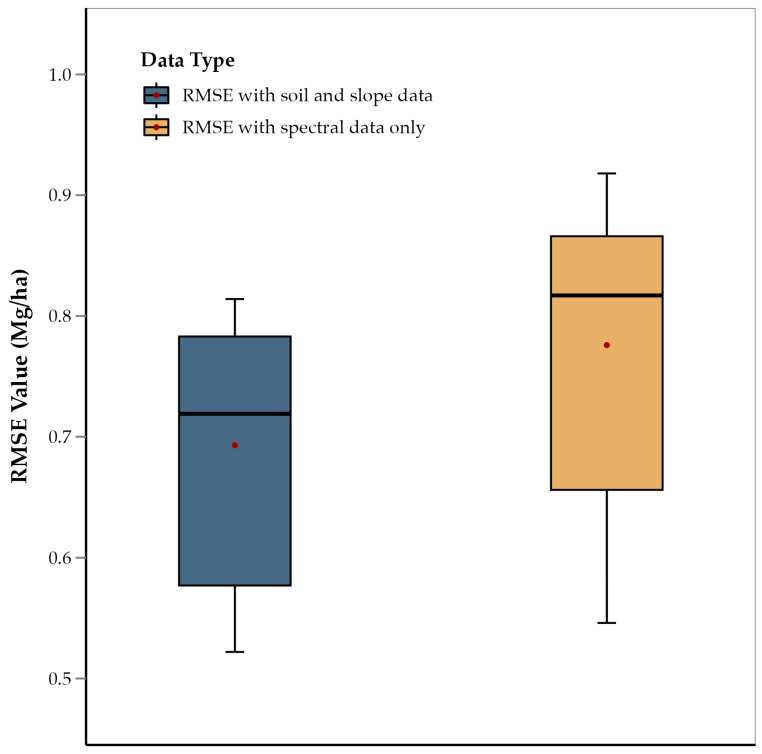
Comparison of average RMSE values of ensemble methods (RF, XGBoost, and GBR) for features including soil and slope data, and features with spectral data only.

The one-tailed *t*-test showed that the average RMSE of those ensemble models that did not consider slope, OM, sand, clay, and silt was significantly higher than models that did include those five predictors (*p* = 0.011) (Figure 6). This suggests that the integration of spectral data with soil composition and slope data improves model accuracy. This is consistent with previous studies on soybean and wheat that used similar data integration techniques for yield prediction [34]. This approach concludes that multiple abiotic factors influence corn yield along with crop physiology, and that integrating diverse data sources captures this complex interaction, as has been observed in other crops, such as soybean, wheat, and sorghum [53,54,55]. Therefore, integration of non-spectral data not only improved results, but in fact, these data were some of the most important predictors. For example: slope, OM, sand, clay, and silt were among the most important features for all growth stages and were present among predictors for all growth stages (Table 2). Variation in soil elevation affects terrain, microclimatic conditions, water drainage, and nutrient distribution, all of which have a direct impact on crop growth and yield [56,58]. Slope affects water drainage and erosion, influencing moisture availability and nutrient distribution across the landscape, which are critical for crop development [59]. OM is a key indicator of soil fertility, contributing to nutrient supply and water retention, both of which are essential for optimal plant growth and yield [58]. Soil texture components, such as sand, clay, and silt, determine the soil’s ability to retain moisture and nutrients, impacting root development and plant health [61]. Sand content influences soil aeration and drainage, while clay and silt enhance the soil’s capacity to hold water and nutrients, creating a balanced environment for root uptake [60]. Therefore, the use of these predictors enhances the model’s ability to account for spatial variability in soil and topographic characteristics, leading to more accurate and reliable yield predictions [104,105,106]. Historically, the need for manual measurements of soil properties and slope might have limited their inclusion in yield prediction models. Collecting such data required time-consuming fieldwork, which was not always feasible. However, with the advent of publicly available high-resolution slope and soil information rasters, such as those from the USGS and POLARIS databases, they can be easily incorporated into yield prediction models. This advancement enables a more efficient and comprehensive approach to data integration.

### 3.5. Leaf Spectral Properties

Based on the final list of predictors used for yield estimation, several indices proved to be crucial. Indices such as GNDRE, TGI, and HUE were present in prediction models of all DAPs, while TVI, ARI, GCI, MRESR, MCARI2, NPCI, RRI, and CCCI appeared in most of the DAPs (Table 2 and Table 3). Among these, GNDRE, which normalizes green and red-edge wavelengths, was used for the first time in this study and emerged as one of the best-performing indices for yield estimation. This can be attributed to the sensitivity of green and red-edge wavelengths to slight changes in leaf chlorophyll [46,107,108]. Leaf chlorophyll (chl) is a primary driver of photosynthesis which can impact yield and is sensitive to reflectance in green (around 560 nm) and red-edge (between 685 nm and 725 nm) bands. Similarly, indices such as MRESR, MCARI2, RRI, and CCCI normalize green and red-edge wavelengths and are some of the best-performing indices for yield estimation. HUE is a measure of color in degrees. Green leaves indicate healthier plants and higher yields; therefore, a shift towards greener leaves would indicate healthy corn, whereas a shift towards yellow would indicate maturity and senescence or plant stress [51,109]. Variations in indices such as NCPI, GCI, ARI, and TGI are influenced by red, green, and blue (RGB) reflectance and are highly correlated with leaf area index, CO_2_ uptake, leaf nitrogen (N), starch content, and leaf pigments such as chl a, chl b, xanthophyll, and carotenoids [15,47]. These pigments are fundamental for light absorption for photosynthesis and are linked to CO_2_ assimilation and synthesis of organic compounds. Higher leaf N content correlates with increased chl production, enhancing photosynthesis and CO_2_ assimilation in the form of starch. Starch accumulates in the corn kernels, directly influencing yield [43,46]. Therefore, we see that these indices capture subtle variations in plant physiology and stress responses, making them valuable tools for precision agriculture.

In our study, we also found that certain Vis, such as NDVI, TrVI, and WDRVI exhibited minimal feature importance across all DAPs (Table S1). These indices are primarily influenced by NIR reflectance, which is highly correlated to leaf cell structure and water content. The NIR reflectance tends to remain relatively stable unless the plants experience extreme biotic or abiotic stress. Since our study did not encounter such extreme stress conditions, the NIR-based indices reached high values early in the season and became saturated. This saturation effect means that these indices were unable to effectively capture the subtle changes in corn growth physiology that occurred throughout the growing season. Previous studies have highlighted such limitations of NIR-dominated indices under non-extreme conditions [110,111]. A study on corn emphasized that while NIR reflectance is useful for detecting overall biomass, it is less sensitive to variations in chlorophyll content and other physiological parameters under typical growing conditions [112].

**Table 2:** Vegetation indices and their formula used in the regression models.

Name	Acronym	Formula	Reference
Enhanced Vegetation Index	EVI	$\frac{2.5\times \left(NIR-Red\right)}{NIR+6\times Red-7.5\times Blue+1}$	[102]
Soil-Adjusted Vegetation Index	SAVI	$\left(1+0.5\right)\times \frac{NIR-Red}{NIR+Red+0.5}$	[113]
Blue Green pigment Index	BGI	$\frac{Blue}{Green}$	[43]
Triangular Vegetation Index	TVI	$\left(0.5\times \left(120\times \left(NIR-Green\right)-200\times \left(Red-Green\right)\right)\right)$	[114]
Modified Chlorophyll Absorption in Reflectance Index (red)	MCARI	$\left(\left(RedEdge-Red\right)-0.2\times \left(RedEdge-Green\right)\right)\times \frac{RedEdge}{Red}$	[44]
Chlorophyll Red-Edge Index	CREI	$\frac{NIR}{RedEdge}-1$	[112]
Plant pigment ratio	PPR	$\frac{Green-Blue}{Green+Blue}$	[46]
Green Chlorophyll Index	GCI	$\frac{NIR}{Green}-1$	[112]
Green Normalized Difference Red Edge Index	GNDRE	$\frac{RedEdge-Green}{RedEdge+Green}$	
Anthocyanin Reflectance Index	ARI	$\frac{1}{Green}-\frac{1}{Red}$	[115]
Canopy Chlorophyll Content Index	CCCI	$\frac{\frac{NIR-RedEdge}{NIR+RedEdge}}{\frac{NIR-Red}{NIR+Red}}$	[116]
Modified Chlorophyll Content Index	MCCI	$\left(\frac{RedEdge-Red}{RedEdge+Red}\right)-\left(\frac{RedEdge-Green}{RedEdge+Green}\right)$	
Simple Ratio	SR	$\frac{NIR}{Red}$	[38]
Normalized Plant Pigment Ratio	NPPR	$\frac{Green}{Blue+Red}$	[15]
Green Atmospherically Resistant Vegetation Index	GARI	$\frac{NIR-\left(Green-\left(Blue-Red\right)\right)}{NIR+\left(Green+\left(Blue-Red\right)\right)}$	[61]
Normalized Pigment Chlorophyll Index	NPCI	$\frac{Red-Blue}{Red+Blue}$	[16]
Visible Atmospherically Resistant Index Green	VARIg	$\frac{Green-Red}{Green+Red-Blue}$	[115]
Enhanced Vegetation Index-rededge	EVIre	$2.5\times \frac{RedEdge-Red}{RedEdge+6\times Red-7.5\times Blue+1}$	
Soil-Adjusted Vegetation Index-rededge	SAVIre	$\left(1+0.5\right)\times \frac{RedEdge-Red}{RedEdge+Red+0.5}$	
Structure Insensitive Pigment Index	SIPI	$\frac{NIR-Blue}{NIR-Red}$	[45]
Modified Red Edge Simple Ratio	MRESR	$\frac{NIR-Blue}{RedEdge-Blue}$	[117]
RedEdge Ratio Index	RRI	$\frac{RedEdge}{Red}$	[118]
Normalized Difference Vegetation Index Red Edge	NDVIre	$\frac{RedEdge-Red}{RedEdge+Red}$	[119]
Modified Chlorophyll Absorption in Reflectance Index (rededge)	MCARI2	$\left(\left(NIR-RedEdge\right)-0.2\times \left(NIR-Green\right)\right)\times \frac{NIR}{RedEdge}$	[108]
Triangular Greenness Index	TGI	$-0.5\times \left(190\times \left(Red-Green\right)-120\times \left(Red-Blue\right)\right)$	[120]
Hue	HUE	$\mathrm{DEGREES}\left(\cos^{-1}\left(\frac{0.5\times \left(\left(\mathrm{Red}-\mathrm{Green}\right)+\left(\mathrm{Red}-\mathrm{Blue}\right)\right)}{\sqrt{{\left(\mathrm{Red}-\mathrm{Green}\right)}^{2}+\left(\mathrm{Red}-\mathrm{Blue}\right)\times \left(\mathrm{Green}-\mathrm{Blue}\right)}}\right)\right)$	[121]

**Table 3:** Final list of predictors used for yield estimation on all respective days after planting (DAP). The predictors are in sequence of feature importance (FI seq) of random forest.

	DAP	20	27	43	55	64	78	83
FI Seq								
1		TVI	MRESR	CCCI	Silt	Sand	TVI	GNDRE
2		MRESR	Clay	Slope	Sand	Silt	NPCI	MRESR
3		SAVI	Sand	Sand	TVI	Slope	TGI	ARI
4		Slope	CREI	Clay	Slope	Clay	Silt	MCARI2
5		Sand	EVI	Silt	Clay	TGI	GCI	CREI
6		Clay	HUE	GCI	TGI	TVI	HUE	Sand
7		Silt	CCCI	ARI	NIR	MCARI	ARI	Clay
8		HUE	Slope	TGI	MCARI	NPPR	Slope	GCI
9		BGI	GCI	MRESR	GNDRE	MCARI2	MCARI2	Silt
10		GARI	RRI	TVI	OM	GCI	MRESR	TGI
11		ARI	TGI	OM	NPPR	ARI	Clay	Slope
12		TGI	Silt	NPCI	ARI	OM	RRI	OM
13		CREI	NIR	HUE	NPCI	GNDRE	Sand	RRI
14		GCI	TVI	MCARI2	MCCI	RRI	OM	NPCI
15		SIPI	GNDRE	GNDRE	RRI	SR	VARIg	CCCI
16		RRI	OM	BGI	HUE	NPCI	SR	MCARI
17		CCCI	MCARI2	PPR		HUE	GNDRE	HUE
18		OM						
19		GNDRE						

### 3.6. Distribution of Measured vs. Predicted Yield

**Figure 7:**
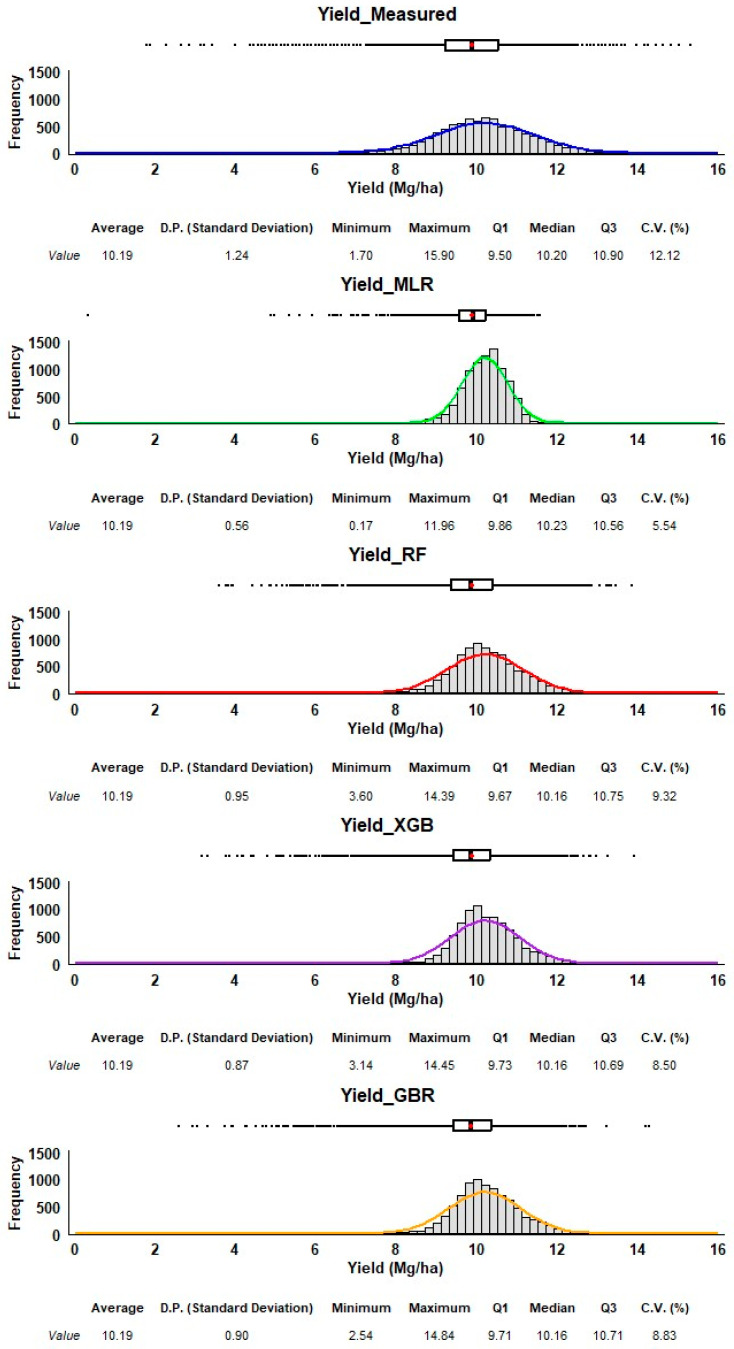
Frequency distribution and summary statistics of measured and estimated corn yields. The estimated corn yield is from the V14/VT stage using all four regression methods (MLR, XGB, RF, GBR). The red dot in the box plot is the average.

Each method’s performance is represented through histograms and boxplots, allowing us to assess their accuracy and precision in predicting corn yield (Figure 7). The measured yield (top) serves as the reference distribution. The data for all predictive methods are relatively symmetrical, with no extreme skew. The measured yield has a bell-shaped distribution, with an average of 10.19 Mg/ha and a relatively small spread (standard deviation of 1.24), and a range from 1.70 Mg/ha to 15.90 Mg/ha. All four methods have an average yield of 10.19 Mg/ha, which matches the measured data. However, the predicted yields vary in their spread around the mean. While RF, GBR, and XGB show standard deviations closer to the measured data (0.90 to 0.95), indicating a broader range of predictions, the MLR method underestimates the variability of yield compared to the measured data, as seen in its much narrower distribution in the histogram. The boxplot confirms this with very little spread and no significant outliers. Both RF and XGB predictions are slightly right-skewed, meaning there are more higher-yield predictions compared to lower ones. GBR has a predicted minimum of 2.54 Mg/ha, which is lower than those of XGB and RF (3.60 Mg/ha and 3.14 Mg/ha, respectively). These minimum values are much higher than the minimum measured yield, suggesting that RF, XGB, and GBR may not predict extremely low yields well. On the other hand, RF, XGB, and GBR have maximum values around 14 Mg/ha, much higher than MLR (11.96 Mg/ha), though some extreme values may still be missing. MLR provides the most tightly grouped predictions, with the smallest variability, but it likely underestimates the actual variability of corn yield. RF, XGB, and GBR all produce predictions with broader distributions, capturing more of the variability observed in the measured data. RF and XGB show some skew towards higher yields, which may indicate a bias toward overestimating higher yields. GBR offers the best balance between variability and matching the range of the measured yield, capturing lower values while still covering the high end of the spectrum. Among the methods, GBR and RF seem to provide more realistic predictions in terms of variability and range. In contrast, MLR may be overly simplistic in its prediction range, as it underestimates yield variability. XGB performs similarly to RF but shows a slight skew that might affect predictions of extreme values. Also, RF and GBR look similar, but RF has better machine learning metrics than GBR; therefore, RF is our recommended method.

## 4. Conclusions

This study demonstrated the effectiveness of using leaf spectral properties along with abiotic factors, such as soil properties and slope, for improving corn yield estimation through machine learning models. The RF model provided the most accurate yield predictions, and its superior performance can be attributed to its robust ability to handle large datasets, to model complex, non-linear interactions among predictor variables, and to avoid overfitting. This capability is particularly advantageous in agricultural settings, where data can be noisy and variable. The study also concluded that the most accurate yield predictions occurred during the V12 to V14/VT stages. These vegetative stages involve significant physiological processes, including pollen formation and nutrient translocation, which directly influence cob growth and seed yield. Additionally, early-season yield estimation (at V5) was found to be feasible, with comparable accuracy to mid-season estimations. This conclusion can help identify optimal UAS flight stages for corn, potentially saving resources and time by reducing the need for multiple flights during vegetative stages. One of the key conclusions of our study was that integrating leaf spectral properties with abiotic factors, such as slope, OM, sand, silt, and clay content, significantly improved model accuracy. Moreover, these non-spectral parameters were some of the best predictors for regression models at all growth stages. Our study determined that indices, such as TGI, HUE, and GNDRE, were significant for yield prediction. These VIs capture important physiological traits of corn, including chlorophyll content, leaf area index, and pigment concentrations, which are crucial indicators of plant health and stress response. While certain indices like NDVI, TrVI, and WDRVI showed minimal feature importance due to their saturation under non-extreme stress conditions, the overall model accuracy benefited from the inclusion of diverse predictors that captured variations in plant physiology and environmental conditions. This finding suggests that future studies should continue to explore the use of diverse spectral and non-spectral data sources to enhance yield prediction models.

Based on our findings, our main recommendation is data integration of abiotic features, such as soil properties and slope, with leaf spectral properties for improving corn yield prediction. This approach complements the predictive ability of leaf spectral features and provides more accurate and reliable results. Among the machine learning methods tested, RF performed the best, handling complex datasets and capturing non-linear interactions effectively. Based on the research findings, optimal flight campaigns should be focused on the V5 and V14/VT stages during the vegetative stages to reduce operational costs. Lastly, we recommend using indices based on RGB combined with red-edge for yield prediction. These indices, such as GNDRE (used for the first time in this study) and TGI, were shown to be highly effective, as they are sensitive to chlorophyll and plant health. While our study successfully utilized multiple VIs, soil properties, and topographic features to predict corn yield, machine learning for yield prediction has certain limitations. The use of more advanced spatial analytics could potentially improve accuracy. Also, incorporating more diverse environmental variables and testing the model across different geographical regions would enhance its robustness and applicability. Future studies should explore the integration of convolutional neural networks and other deep learning techniques to optimize the use of multispectral data by combining VIs and individual bands. Overall, the findings from this study highlight the potential of combining UAS-based remote sensing with advanced data analytics to enhance precision agriculture and improve crop management strategies. Conducted in a rain-fed field with minimal management disturbances, the research utilized high-resolution multispectral data, vegetation indices, and advanced models to predict yields with notable accuracy. By incorporating soil and topographic variability, the models achieved robust performance, including early-stage yield estimation at the V5 growth stage.

In agriculture, machine learning offers innovative solutions to improve decision-making, enhance crop yield predictions, optimize resource use, and better understand environmental factors affecting farming practices. Machine learning models can detect patterns, predict outcomes, and identify correlations that may not be immediately apparent through conventional methods. This approach enables farmers to make data-driven decisions that support precision farming, regenerative practices, and promoting sustainability. Therefore, it is crucial to present these applications clearly, demonstrating how the machine learning models were trained, validated, and applied to the agronomic context, and how they can lead to practical, real-world improvements.

## Figures and Tables

**Table 1:** Date of flight campaign and its corresponding corn phenology and days after planting (DAP).

Date of Flight	Corn Phenology	DAP
March 20th	4-leaf stage (V4)	20
March 27th	5-leaf stage (V5)	27
April 12th	6-leaf stage (V6)	43
April 24th	7-leaf stage (V7)	55
May 3rd	9-leaf stage (V9)	64
May 17th	12-leaf stage (V12)	78
May 22nd	14-leaf/tasseling stage(V14/VT)	83
-	Harvest	167

## Data Availability

The data and code used in this study are available upon request from the corresponding author. Given that the study involves large volumes of aerial images and derived rasters, we are currently seeking a repository that can accommodate high-volume datasets.

## Supplementary Materials

The following supporting information can be downloaded at https://www.mdpi.com/article/10.3390/s25020543/s1. Supplementary Table S1: Vegetation indices and their formula which were not included in the final models (because they ranked lower in feature importance list of RF model). Refs [122,123,124,125,126,127,128,129,130,131,132,133,134] cited in Supplementary Materials.
