# Supplementary material for: Integrating Remote Sensing and Soil Features for Enhanced Machine Learning-Based Corn Yield Prediction in the Southern US
Source: Sensors (Basel). 2025 Jan 18;25(2):543. doi: 10.3390/s25020543 (PMC11769266; doi:10.3390/s25020543)
Supplement: Supplementary file 1 [file sensors-25-00543-s001.zip › sensors-3401191-supplementary.pdf]

Supplementary Table S1: Vegetation indices and their formula which were not included in the final models (because they ranked lower in feature importance list of RF model).

Name	Acronym	Formula	Reference
Normalized Difference Vegetation Index	NDVI	$\frac{NIR - Red}{NIR + Red}$	[122]
Green Normalized Difference Vegetation Index	GNDVI	$\frac{NIR - Green}{NIR + Green}$	[42]
Normalized Green Red Difference Index	NGRDI	$\frac{Green - Red}{Green + Red}$	[38]
Wide Dynamic Range Vegetation Index	WDRVI	$\frac{0.1 \times NIR - Red}{0.1 \times NIR + Red}$	[123]
Normalized Difference Red Edge Index	NDRE	$\frac{NIR - RedEdge}{NIR + RedEdge}$	[115]
Modified Soil-Adjusted Vegetation Index	MSAVI	$\frac{2 \times NIR + 1 - \sqrt{(2 \times NIR + 1)^2 - 8 \times (NIR - Red)}}{2}$	[124]
Transformed Soil-Adjusted Vegetation Index	TSAVI	$\frac{0.5 \times (NIR - 0.5 \times Red - 0.5)}{Red + 0.5 \times NIR - 0.5 \times 0.5 + 1}$	[125]
Plant Senescence Reflectance Index	PSRI	$\frac{Red - Blue}{RedEdge}$	[126]
Transformed Vegetation Index	TrVI	$\sqrt{\frac{NIR - Red}{NIR + Red} + 0.5}$	[122]
Green Leaf Index	GLI	$\frac{2 \times Green - Red - Blue}{2 \times Green + Red + Blue}$	[127]
Modified Triangular Vegetation Index	MTVI	$1.2 \times \big(1.2 \times (NIR - Green) - 2.5 \times (Red - Green)\big)$	[106]
Chlorophyll Absorption Ratio Index	CARI	$\left(\frac{RedEdge}{Red}\right) \times (Red \times 1.001047 - 0.01585)$	[128]
Plant Phenology Index	PPI	$-0.442 \times \ln\big(0.92 - (NIR - Red)\big)$	[129]
Green-Blue Normalized Difference Vegetation Index	GBNDVI	$\frac{NIR - (Blue + Green)}{NIR + (Blue + Green)}$	[130]
Visible Atmospherically Resistant Index rededge	VARIre	$\frac{RedEdge - 1.7 \times Red + 0.7 \times Blue}{RedEdge + 2.3 \times Red - 1.3 \times Blue}$	[116]
Red-Blue Normalized Difference Vegetation Index	RBNDVI	$\frac{NIR - (Red + Blue)}{NIR + (Red + Blue)}$	[130]
Green-Red Normalized Difference Vegetation Index	GRNDVI	$\frac{NIR - (Red + Green)}{NIR + (Red + Green)}$	[130]
Leaf Chlorophyll Index	LCI	$\frac{NIR - RedEdge}{NIR + Red}$	[131]
Red green ratio	RGR	$\frac{Red}{Green}$	[132]

Excess Green Index	ExGI	$2 \times Green - Blue - Red$	[133]
Saturation	SAT	1. $1 - \left(\frac{\min(Red+Green+Blue)}{\max(Red,Green,Blue)}\right)$	[121]
Intensity	INT	$\frac{Red + Green + Blue}{\sqrt{3}}$	[121]
Red Edge Inflection Point	REIP	$700 + 40 \times \frac{RedEdge - Red}{NIR - Red}$	[52]
Red-Edge Position Linear Interpolation	REP	$700 + 40 \times \frac{(Red + RedEdge)/2 - Red}{NIR - Red}$	[134]
